# Hormonal Contraceptive Use in Football Codes in Australia

**DOI:** 10.3389/fspor.2021.634866

**Published:** 2021-02-25

**Authors:** Anthea C. Clarke, Georgie Bruinvels, Ross Julian, Pip Inge, Charles R. Pedlar, Andrew D. Govus

**Affiliations:** ^1^Sport and Exercise Science, School of Allied Health, Human Nutrition, and Sport, La Trobe University, Melbourne, VIC, Australia; ^2^Orreco Ltd., Business Innovation Centre, National University of Ireland Galway, Galway, Ireland; ^3^Faculty of Sport, Health and Applied Science, St Mary's University, Twickenham, United Kingdom; ^4^Department of Neuromotor Behavior and Exercise, Institute of Sport Science, Westfaelische Wilhelms-University Muenster, Münster, Germany; ^5^School of Sport and Exercise, Exercise and Sport Research Centre, University of Gloucestershire, Cheltenham, United Kingdom; ^6^Australian Football League, Docklands, Melbourne, VIC, Australia; ^7^Division of Surgery and Interventional Science, Institute of Sport, Exercise and Health, University College London, London, United Kingdom

**Keywords:** oral contraception, physiology, female athlete, women, elite sport

## Abstract

The recent launch of the new National elite women's football competitions in Australia has seen a 20–50% increase in grassroots female participation. With the growing participation across grassroots to elite competitions, understanding the health of female athletes should be prioritized. In elite level athletes, hormonal contraceptive (HC) use is common (~50%), however, little is known about the prevalence and reasons for use and disuse of HC in elite female football athletes. As such, the impact of HC use is often not considered when monitoring the health of female footballers. This study involved a subset of data collected as part of a larger questionnaire investigating menstrual cycle function, hormonal contraception use, and the interaction with training load volume and perceived performance in elite female football code athletes. A total of 177 participants completed the questionnaire across three football codes within Australia (rugby league, rugby union/sevens, Australian football). One third (*n* = 58) of athletes were currently using HC, predominately in the form of an oral contraceptive pill (OC, *n* = 47). Reasons for use included: to avoid pregnancy (71%); to control/regulate cycle (38%); and to reduce menstrual pain (36%). However, most athletes using an OC (89%) could not identify the type of pill used (e.g., mono-, bi-, or triphasic). The main reason for disuse was due to the negative side effects (*n* = 23), such as mood swings, weight gain, and depression/anxiety. Comparing HC users and non-users, there were no statistical differences in the number of reported menstrual symptoms, use of medication to relieve menstrual pain, or frequency for needing to adapt training due to their menstrual cycle (*p* > 0.05). Since most athletes were unaware of the type of OC they used, female football athletes require further education about the different types of HC, and specifically OC, available to them. Similarities in the symptoms experienced, pain management, and training adaptation requirements between groups suggests that HC use may not have the intended outcome for certain athletes. As such, greater awareness of athlete's personal experiences with the menstrual cycle, how HC may influence their experience, and acknowledgment of non-pharmacological methods to help manage menstrual cycle related symptoms are warranted.

## Introduction

Monitoring the menstrual cycle for athlete's optimal health and performance is now recognized as an important aspect of female sport (Harber, 2011). A recent meta-analysis looking at the influence of menstrual cycle phase on exercise performance highlights the challenges in clearly identifying this relationship due to poor quality studies and variation in study design (McNulty et al., 2020). Given these challenges, it is recommended that individualized approaches to managing an athletes' training across their menstrual cycle be taken (McNulty et al., 2020). While research is inconclusive as to whether menstrual cycle phase affects performance, many athletes report negative symptoms and feel that they perform worse at certain phases of their cycle (Armour et al., 2020). To alleviate these symptoms, oral contraceptive (OC) use has been reported as common practice among athletes (Schaumberg et al., 2018).

Understanding the prevalence of hormonal contraceptive (HC) use and their reasons for use/disuse are important for practitioners to develop appropriate monitoring and management practices for female athletes. Approximately 50% of elite British athletes use HC (Martin et al., 2018), while similar values are reported in elite Australian athletes (47%, Larsen et al., 2020). In recreational to elite level Australian athletes, the use of HC use is slightly lower at ~40% (Armour et al., 2020), likely due to the inclusion of recreational level participants. Despite the advantages of using HC to control their menstrual cycle and potentially reduce menstrual-related symptoms, evidence suggests potential negative outcomes associated with HC use such as a higher risk for depression (Anderl et al., 2020), lower bone mineral density (Allaway et al., 2020), and greater oxidative stress (Cauci et al., 2016), particularly with OC methods. Understanding the reasons why athletes use HC is important to inform athletes' decision to start, stop, or switch between different HC options.

The number of elite women's National-level competitions, specifically within the football codes of Australian football, rugby union, and rugby league has increased over the past 10 years. While these athletes are considered to be playing at the top-level of competition for their sport, given the infancy of these competitions, little research is available on these athletes to inform athlete management practices. Additionally, most research regarding the menstrual cycle and hormonal contraceptive use to date has been conducted on the general population (Schaumberg et al., 2018; Mackay et al., 2019; Freemas et al., 2020) or endurance athletes (Redman and Weatherby, 2004; Solli et al., 2020). Further research is therefore necessary to report the prevalence and reasons for HC use within female football codes to help shape athletes' management and monitoring practices. The aim of this study was to determine the prevalence and reasons for HC use in athletes competing in female football codes in Australia.

## Materials and Methods

### Questionnaire Design

The data used in this study are a subset from a larger questionnaire completed between September 2019 and May 2020, hosted on Qualtrics and adapted from previously used menstrual cycle questionnaires (Armour et al., 2020; Bruinvels et al., 2020). The larger questionnaire gathered data from six areas; sporting background, menstrual cycle function, medical history, hormonal contraceptive use, the interaction between training loads and the menstrual cycle, and education and communication practices regarding the menstrual cycle. This study primarily used data from the HC use section, sporting background (for participant characteristics), and questions regarding menstrual-related symptoms, use of medication to alleviate symptoms, and the need to adapt training. All questions used within this study were multiple choice answers, with some questions allowing an option for “Other, please specify.” This project was approved by the La Trobe University Human Ethics committee (HEC19066). Participants provided informed consent and the questionnaire was conducted anonymously.

### Recruitment Strategy

Participants were eligible to complete the survey if they were currently competing in a female football code at a representative level and were 16 years of age or over at the time of completing the survey. National sporting bodies were contacted via email and asked to distribute the survey to their player network. The following sporting bodies approved the questionnaire to be disseminated to their state and national representative players: Australian Football League, Rugby Australia, National Rugby League.

Within Australia, there are an estimated 420 elite female Australian football players, 90 elite rugby league players, 100 elite rugby union players, and 150 elite rugby sevens players (although some athletes may play in both rugby union and rugby sevens competitions, and so the total number of elite athletes across both sports is likely lower). To be considered a representative sample, the aim was for a minimum 20% response rate within each sport.

### Statistical Analysis

Descriptive statistics are presented as mean (standard deviation) for normally distributed data and counts and proportions (%) for categorical data. Chi-squared analysis was used to compare responses between hormonal contraceptive and non-hormonal contraceptive groups, with statistical significance set at *p* ≤ 0.05.

## Results

The 20% response rate was achieved for each sport, with 177 athletes responding to the survey. Respondent characteristics are presented in Table 1. One third of respondents (*n* = 58) were currently using a HC at the time of survey completion. Most respondents were participating in 4–7 h (*n* = 91, 51%) or 8–12 h (*n* = 61, 34%) of field-based training, and 1–3 h (*n* = 82, 46%) or 4–7 h (*n* = 87, 49%) of gym-based training per week.

**Table 1:** Participant characteristics of HC and non-HC users. Data presented as mean (SD) or number; percentage of total within sport^a^ or HC-user group^b^.

	**HC users (*n* = 58)**	**Non-HC users (*n* = 119)**	**Total (*n* = 177)**
Age (y)	24.9 (5.2)	24.3 (7.2)	24.5 (5.2)
Height (m)	1.68 (0.06)	1.71 (0.32)*	1.70 (0.07)
Mass (kg)	71.5 (12.5)	71.0 (15.7)	71.1 (11.6)
Sport (*n*; %)^a^			
Rugby League	17; 35%	32; 65%*	49; 24%
Rugby Union/Sevens	22; 46%	26; 54%	48; 24%
Australian Football	19; 24%	61; 76%*	80; 39%
Age at menarche (*n*; %)^b^			
11 years or younger	9; 16%	13; 11%	22; 12%
12–14 years	34; 59%	77; 65%	111; 63%
15 years or older	15; 26%	26; 22%	41; 23%
Don't remember	0; 0%	3; 3%	3; 2%
Duration competing at current level (*n*, %)^b^			
0–2 years	28; 48%	60; 50%	88; 50%
2–4 years	21; 36%	39; 33%	60; 34%
4–6 years	5; 9%	9; 8%	14; 8%
>6 years	5; 9%	17; 14%*	22; 12%

a *Percentage represented from total within each specific sport*.

b *Percentage represented from total within hormonal contraceptive use category*.

*HC, hormonal contraceptive*.

* *Significant difference between HC and non-HC groups; p < 0.05*.

**Figure 1:**
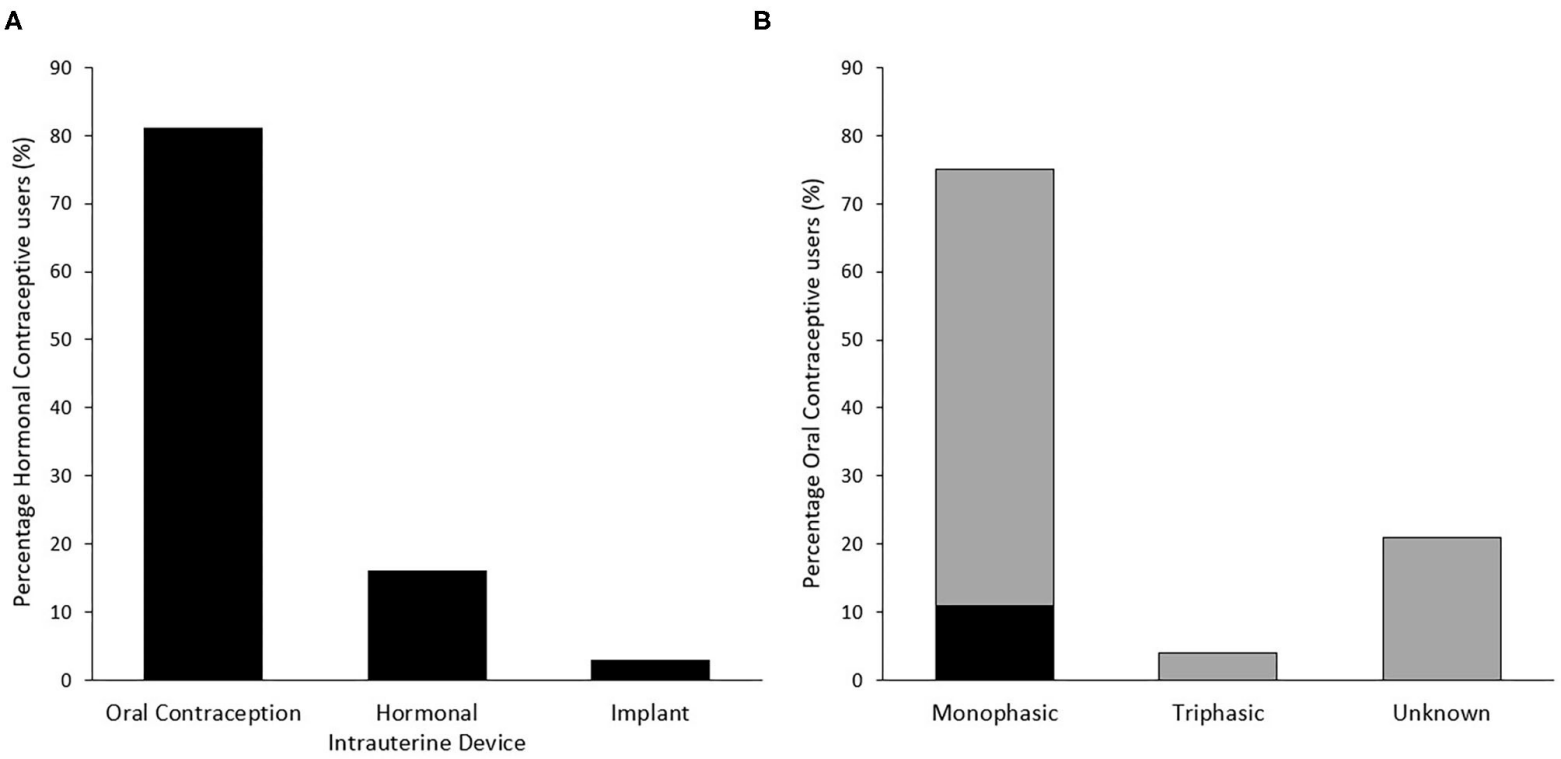
Distribution of type of hormonal contraceptive use (*n* = 58) **(A)** and specific type of oral contraception used (*n* = 47) **(B)** among respondents. Gray bars in panel B indicate an initial response of “Unknown,” of which some responses were subsequently able to be classified by researchers based on identification of specific brand used.

**Figure 2:**
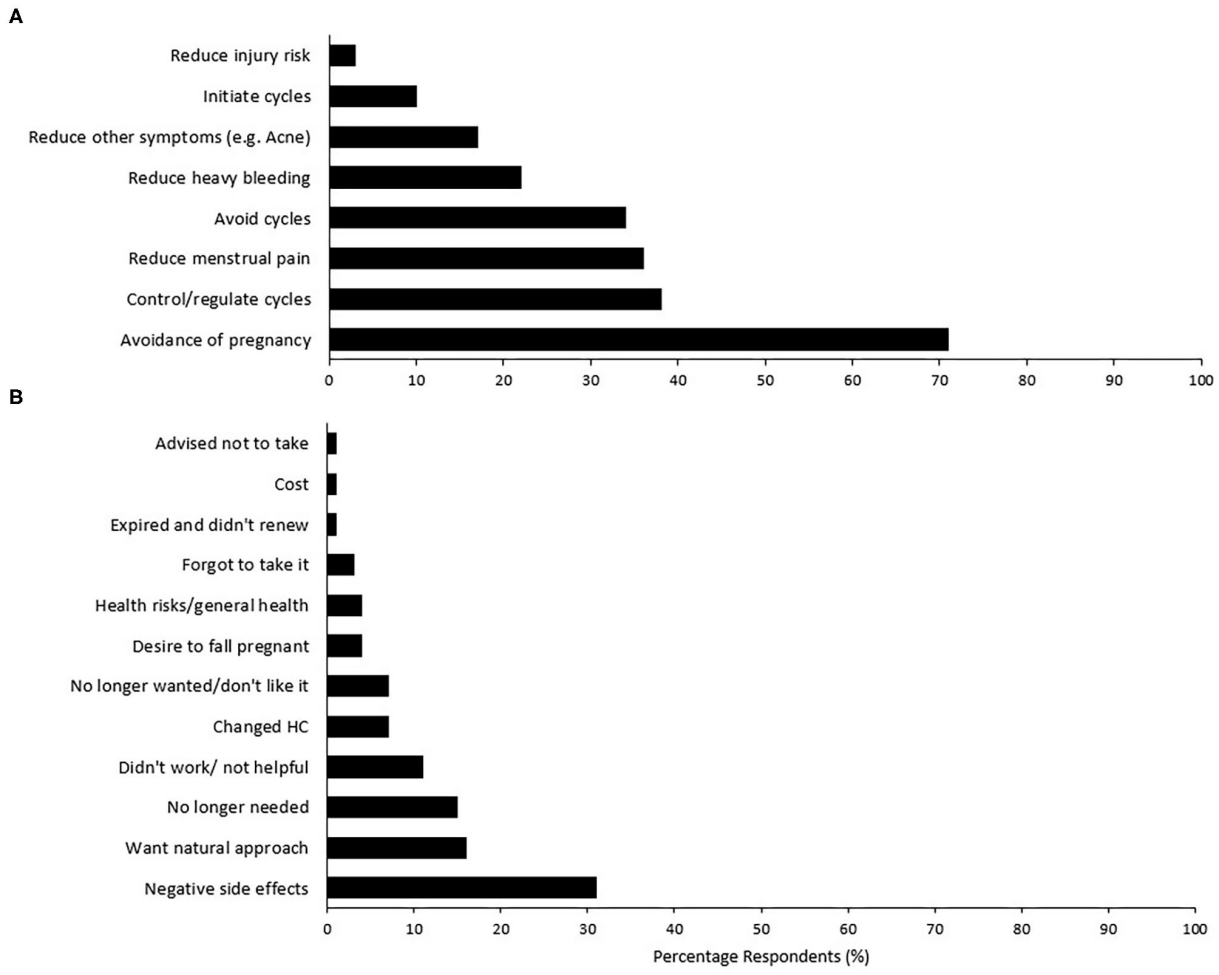
Percentage respondents for **(A)** reasons for hormonal contraception use in current users (*n* = 58), and **(B)** reasons for no longer taking a previous form of hormonal contraception (*n* = 73). Participants were able to select all reasons that apply for current use, while reasons for no longer using was an open-ended question that was subsequently grouped by theme.

The types of HC used and the specific types of OC used are presented in Figure 1. The majority of players using OC could not identify the type of pill used (i.e., monophasic, triphasic, *n* = 42, 89% of OC users). Of those who could not identify the type, 10 (21% of OC users) were also unable to identify the brand of OC pill used. HC use for greater than 5 years accounted for the largest group of users (*n* = 22, 38%), while 13 (22%) had used their current HC method for 12 months or less. Reasons for using a hormonal contraceptive included: to avoid pregnancy; to control/regulate their cycle; and to reduce menstrual-related pain (Figure 2A). Most users (*n* = 23, 40%) reported experiencing no side effects of using a HC, while light or no periods (*n* = 18), mood fluctuations (*n* = 13), and weight gain (*n* = 10) were the most commonly reported side effects for others.

Previous use of HC was reported in 73 (41%) respondents, across Australian football (*n* = 33), rugby union/sevens (*n* = 22), and rugby league (*n* = 18). Fifty-seven of these respondents no longer use any HC, while 16 replaced their previous HC use with a different HC type. Of those who changed HC, eight changed from one OC to another (often with time between use), three changed from an OC to a hormonal intrauterine device, two from an OC to an implant, two from an implant to an OC, and one from an implant to a hormonal intrauterine device.

**Figure 3:**
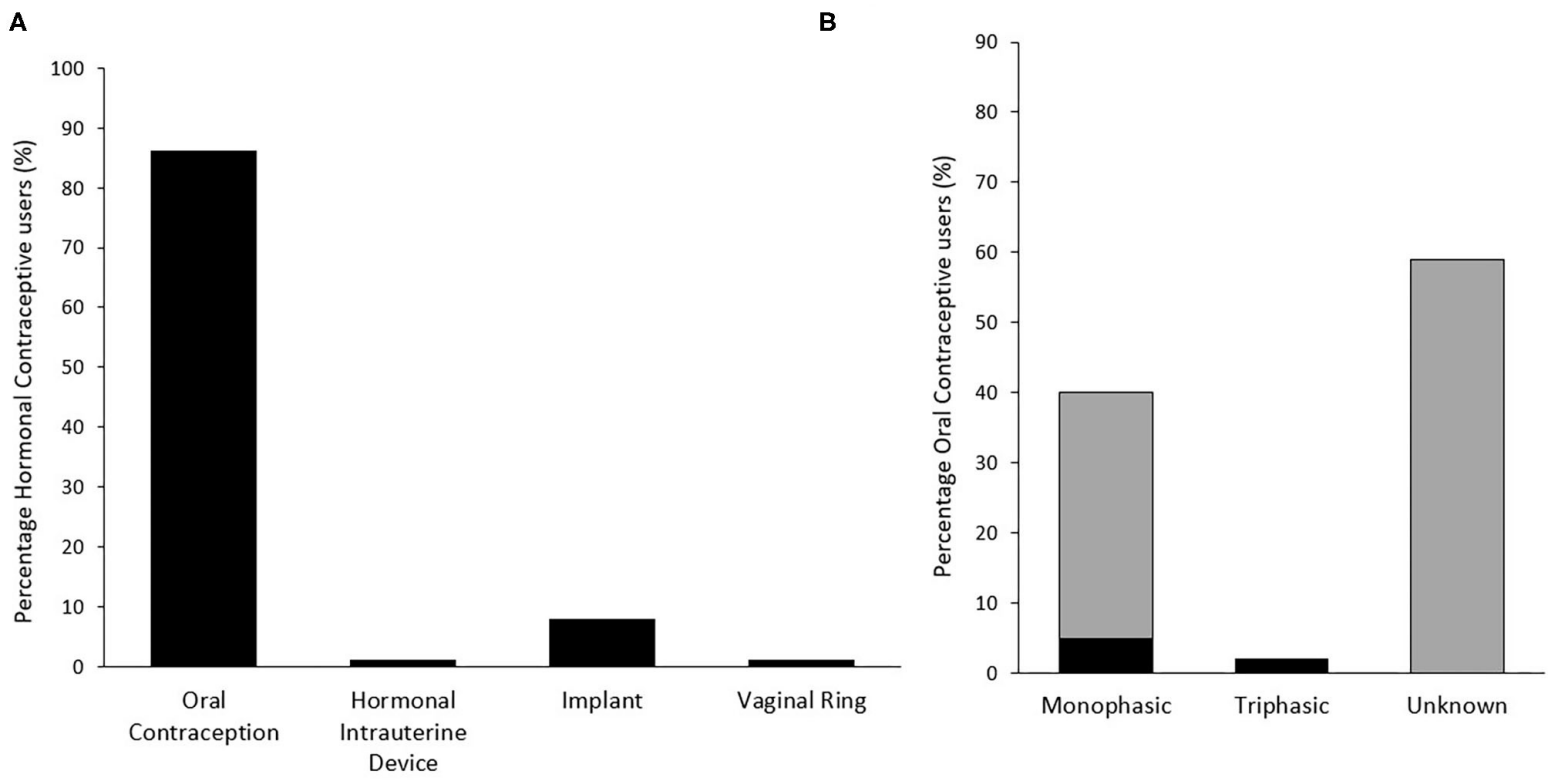
Distribution of previously used hormonal contraceptive type (*n* = 73) **(A)** and specific type of oral contraception used (*n* = 63) (**B**) among respondents. Gray bars in panel B indicate an initial response of “Unknown,” of which some responses were subsequently able to be classified by researchers based on identification of specific brand used.

The types of HC previously used and the specific types of OC are presented in Figure 3. The reasons for no longer using this form of contraception are presented in Figure 2B. The 57 respondents who previously used a HC, but no longer do, are made up of athletes from Australian football (*n* = 29), rugby union/sevens (*n* = 15), and rugby league (*n* = 13). Negative side effects (*n* = 23) was the main reason for discontinuation, including mood swings, weight gain, depression/anxiety, and headaches/migraines. Eleven individuals cited that it was no longer needed, however, only five specified the reason why—minimal sexual activity (*n* = 2), acne under control (*n* = 2), and sexual orientation (*n* = 1). Mean duration of previous HC use was 3.5 ± 3.3 years.

Across all respondents, only nine athletes reported not experiencing any pre-menstrual symptoms, of which three were HC users. For the remaining respondents, a mean of 6 ± 4 different symptoms were reported, with the most common symptoms being stomach cramps (*n* = 116) and changes in mood (*n* = 100). There was no statistical difference (*p* > 0.05) in the number of symptoms experienced by HC users and non-users. Overall, use of medication to treat menstrual-related symptoms was reported by 69 athletes (32%), while five athletes chose not to respond. While not statistically different, within hormonal contraceptive users, 20 (34%) required the use of medication to alleviate menstrual-related symptoms, compared to 49 (41%) of non-HC users. Due to menstrual related symptoms, training was missed (*n* = 31, 18%) or had to be adapted (*n* = 56, 32%) by athletes typically 1–5 times per year. The number of athletes who reported missing training or competition due to menstrual related symptoms was similar in HC (*n* = 9, 16%) and non-HC users (*n* = 22, 18%), as was the need to adapt training (HC users, *n* = 15, 26%; non-HC users, *n* = 41, 34%).

## Discussion

Approximately one third of athletes from the three codes of football in Australia currently use a form of HC, most commonly a monophasic OC pill. However, almost 90% of OC users could not identify the specific type that they use. The most common reasons for use of HC were (1) to prevent pregnancy, (2) to control their cycle, and (3) to reduce menstrual-related pain. There was no difference between HC users and non-users in relation to menstrual symptoms experienced, use of medication, and the need to adapt or miss training due to their menstrual cycle.

This study observed a lower HC use among elite female football athletes—approximately one third (32.7%)—compared to research conducted in recreational to elite Australian athletes (42%, Armour et al., 2020) and elite Australian (47%, Larsen et al., 2020), British (50%, Martin et al., 2018), and Danish (57%, Oxfeldt et al., 2020) athletes across both team and individual sports. Given a limited amount of research has investigated menstrual cycle and HC use in team sport athletes, it is unknown whether the lower prevalence of HC use in the current study is sport related, or a result of sociocultural, geographical, or other influencing factors. Given several athletes in this cohort have previously used an HC, and the second most common reason for discontinuation was that they wanted to take a “natural” approach, understanding the changing attitudes toward the use of HC may be an important consideration. There is a growing body of literature to suggest negative effects such as depression (Anderl et al., 2020), poorer bone mineral density (Allaway et al., 2020), and greater oxidative stress (Cauci et al., 2016) with HC use. However, these effects may be specific to the HC type, with OC the main focus of research looking at these such side effects. The use of HC also masks the natural fluctuations in endogenous hormones, limiting the ability to use the menstrual cycle as an indicator of general health (Harber, 2011). Further, for those athletes who choose to discontinue HC use, it is important for both athletes and their coaching staff to recognize that menstrual disturbances may be present for up to 9 months following cessation (Gnoth et al., 2002). Assessment of athletes' perceptions of HC use for health and performance reasons would be beneficial to understanding the factors contributing to HC use and disuse. Understanding where athlete's source their information regarding this choice and the specific experiences of athletes as they transition away from HC use may also be beneficial to enable the optimal management of female athletes.

Many of the athletes surveyed were unable to specify the type or brand of OC they used, suggesting either a lack of interest in, or awareness of, the different types and/or benefits and risks of OC use. Research in Australian athletes has suggested a poor knowledge in relation to the menstrual cycle and use of HC (Larsen et al., 2020). Greater education may be needed for athletes to make informed choices about their HC use, in conjunction with their medical doctor, to ensure optimal health, well-being, and performance. It is important that athletes understand that an individual approach to choosing a HC is required and they are not guided by other teammates or team coaches, given each has a unique experience with HC and their menstrual cycle.

We did not observe any differences between HC users and non-users in relation to experiencing various menstrual-related symptoms (with both groups reporting stomach cramps and changes in mood as the most common symptoms), the need to use medication, or having to adapt training due to these symptoms. This is despite reasons for using HC including symptom management and reducing menstrual pain. Reported negative symptoms are also similar between HC users and non-users in elite Danish athletes, whereas positive symptoms were reported more frequently in non-HC users (Oxfeldt et al., 2020). However, this research only asked to identify the types of symptoms experienced, rather than rate their severity or duration. We speculate that it is possible that while HC users still experience these symptoms, the severity or duration may be lower than compared to what they would experience if they were not using a HC. Alternatively, HC have been associated with greater inflammation and oxidative stress (Cauci et al., 2016), which may exacerbate pre-menstrual symptoms in some individuals. Further research that looks at both endogenous and exogenous hormones during HC use may help gain further insight in understanding the etiology of symptom manifestation.

This research is the first to look at athletes' experiences and reasons for HC use and disuse in elite football codes in Australia. With the growing professionalism for women's teams within these codes, understanding this information is important to assist with the management of athletes' health and well-being. However, it is important to recognize that these findings are specific to this population group, and other elite women's team sports may have different findings specific to their sport or geographic location, based on different medical advice or personal preferences. The ~20% response rate (based on the estimated total population size) may also limit the generalizability of these findings, hence further work is required to corroborate these findings. Participants' involvement in this study may also be influenced by self-selection bias. Consequently, participants who completed the study may have already been interested in, or aware of, how menstrual cycle and HC use interacts with their health, training, and performance. Additionally, this research focused on elite players, however, the practices of athletes competing in sub-elite, youth, and recreational competitions may be more diverse due to a different emphasis on general health vs. performance, or based on the resources available to them within their sporting environment. Determining what factors contribute to athletes deciding to use (or stop using) HC, and the specific types and formulations, is an important avenue for future research in female athletes. Understanding the transition between exogenous (HC use) and natural hormones stabilizing following HC discontinuation, and the subsequent influence on menstrual-related symptoms and performance is also an important consideration for practitioners and the need for individualized athlete management. This will ensure sporting bodies and associated medical professionals are able to specifically target the education and management practices for female athletes. Whether an athlete's decision is based on health, practicality, science, or sociological influences (e.g., friend/teammate recommendations or experiences) is yet to be examined. This may be important when it comes to how information is received and acted on by athletes, and how sporting clubs can play their role to optimize the health, performance, and well-being of athletes.

Overall, we make three recommendations from this research. First, there is a need for greater education about the menstrual cycle and HC options for athletes. Second, this education should extend to coaches, support staff, and clinicians to ensure the optimal management of female athletes' health and performance. Given Australian athletes do not often discuss their menstrual cycle or related symptoms with coaching staff (Armour et al., 2020), empowering coaches with this knowledge may help to remove any perceived barriers to having these important conversations. Third, further research is required to understand in greater detail the HC experiences of athletes, how HC use influences the performance and recovery of athletes, and explore alternative methods to HC for the management of MC symptoms without negative side effects.

## Data Availability Statement

The raw data supporting the conclusions of this article will be made available by the authors, without undue reservation.

## Ethics Statement

The studies involving human participants were reviewed and approved by La Trobe University Human Ethics Committee. The participants provided their written informed consent to participate in this study.

## Author Contributions

All authors were involved in the study design and questionnaire creation. AC recruited the participants and conducted the statistical analysis. All authors contributed to the writing of the manuscript and reviewed and approved the final draft for submission.

## Conflict of Interest

GB and CP were employed or consultants with the company Orreco Ltd. The remaining authors declare that the research was conducted in the absence of any commercial or financial relationships that could be construed as a potential conflict of interest.
